# *Synorthocladiusfedericoi* sp. nov., a new species occurring in the middle basin of the Adige River, northern Italy (Diptera, Chironomidae, Orthocladiinae)

**DOI:** 10.3897/zookeys.1057.68175

**Published:** 2021-08-27

**Authors:** Valeria Lencioni, Joel Moubayed

**Affiliations:** 1 Department of Invertebrate Zoology and Hydrobiology, MUSE-Museo delle Scienze, Corso del Lavoro e della Scienza 3, 38122 Trento, Italy Museo delle Scienze Trento Italy; 2 Freshwater and Marine biology, 10 ruez des Fenouils, 34070 Montpellier, France Freshwater and Marine biology Montpellier France

**Keywords:** Adige River, Alps, chironomids, orthoclads, adult male, morphology

## Abstract

An adult male *Synorthocladius* was collected in the middle basin of the Adige River in the city of Verona, northern Italy. A combination of atypical characters for the genus signalled a new species. *Synorthocladiusfedericoi***sp. nov.** is here diagnosed and described. The new species is known only from its type locality and is presumed to be a local biogeographical representative of the Italian Pre-Alps. An emended generic diagnosis, a key to known *Synorthocladius* from Europe and comments on the taxonomic position of the new species are given.

## Introduction

According to data on the taxonomy and geographical distribution of known *Synorthocladius* species from the Palaearctic and neighbouring biogeographical regions (Pankratova 1970; Sasa and Yamamoto 1977; Sasa 1981; Cranston et al. 1989; Reiss 1989; Evrard 1995; Liu and Wang 2005; Langton and Pinder 2007; Lencioni et al. 2011, 2012, 2018; Ashe and O’Connor 2012; Moubayed-Breil and Ashe 2012; Plociennik and Pesic 2012; Sæther and Spies 2013; Plociennik and Karaouzas 2014; Makarchenko and Makarchenko 2017; Kettani and Moubayed-Breil 2018; Murray et al. 2018; Yavorskaya et al. 2018; Rossaro et al. 2019; Moubayed-Breil 2020), there are eight valid species of the genus worldwide, of which only one, *S.semivirens* (Kieffer, 1909), was reported from Europe. The present description of *S.federicoi* sp. nov. increases the total number valid species of *Synorthocladius* from Europe to two.

The emended generic diagnosis of the genus given in Cranston et al. (1989) and Liu and Wang (2005) is reviewed and supplemented with some additional characters found in the adult male of the new species.

## Material and methods

The studied adult male was collected using a light trap along the banks of the Adige River (altitude = 61 m a.s.l.; 45°26'58.68"N, 10°58'52.81"E), preserved in 80% ethanol and cleared of musculature in 90% lactic acid for about 70 minutes. When clearing was complete, the material was washed in two changes of 50–60% ethanol to ensure that all traces of lactic acid were removed. It was then mounted in polyvinyl lactophenol. Before the final slide mount (dorsal), the hypopygium including tergite IX, the anal point, the gonocoxite and the gonostylus were viewed ventrally and laterally and all morphological details were drawn from all sides. The rest of the abdomen was preserved in 85% ethanol for possible future DNA analysis. Terminology and measurements follow those of Sæther (1980) and Langton and Pinder (2007). Taxonomic remarks and comments on the ecology of the new species are provided.

## Taxonomy

### Genus Synorthocladius

Taxon classificationAnimaliaDipteraChironomidae

Thienemann, 1935: emended generic diagnosis

C2AE29F8-E0FC-5289-AFB4-26F8BD805AAE

#### Remarks.

The generic diagnosis of *Synorthocladius* in Thienemann (1935), emended in Cranston et al. (1989) and Liu and Wang (2005), is here supplemented as follows. ***Head***: Frontal tubercles present, circular or triangular; coronal triangle reduced or weakly developed; coronal setae present or absent; sensilla coeloconica on palpomere 3 present or absent. Antenna. Last flagellomere simply clubbed, or with bilobed or truncate apex; antennal ratio between 0.5 and 1.0. ***Thorax***: Acrostichals 0–3, or about 9; scutellum with 2 or 4–6 setae; sensilla chaetica present on tibiae and tarsomeres ta_1_–ta_5_ of PI–PII, absent on tarsomeres of PIII. ***Abdomen***: Tergite IX with or without a dorsal hump; anal point slightly to strongly curved upwards. Gonocoxite generally with slender dorsal and ventral inner margin, distinctly broad at base. Virga absent or well developed. Superior volsella flat or broadly swollen. Apex of inferior volsella single or double, long nose-like, lobe-like or truncate, subtriangular or spherical. Gonostylus generally slender to well developed, or atypically globular or bean-shaped as in the new species.

### Synorthocladius federicoi sp. nov.

Taxon classificationAnimaliaDipteraChironomidae

64209269-E0C9-5629-BE13-4CAF0EBA1E6E

http://zoobank.org/886D9D10-C7F4-4B3D-AA73-CE96DCF7C5E9

#### Material examined.

***Holotype***: adult male, leg. L. Latella; Adige River in the city of Verona, Veneto Region, Italy (altitude = 61 m a.s.l.; 45°26'58.68"N, 10°58'52.81"E); 13 April 2020.

The holotype (on one slide and abdomen in one tube) is deposited in the entomological collection of MUSE-Museo delle Scienze, Trento, Italy (Accession number: cINV0017_s61v73).

#### Etymology.

The new species is named ‘*federicoi*’ after Federico, the first author’s son, who has an inherited passion for insects and contributed to the collection of chironomids with the light trap.

#### Diagnostic characters.

***Head***: Frontal tubercles broadly semi-circular, coronal triangle and coronal suture reduced, coronal setae absent; temporals 6; last flagellomere of antenna bilobed apically, with numerous curved sensilla chaetica; AR 0.90. Palpomere 3 without sensilla coeloconica. Clypeus inverted safety helmet shaped, with 5 setae. ***Thorax***: Lobes of antepronotum not gaping, thinner basally; acrostichals 2; dorsocentrals 7–8, uniserial; prealars 4; humeral pit absent; scutellars 6; squama with 4–5 setae. Legs. Sensilla chaetica on tibiae and tarsomeres ta_1_–ta_5_ of PI–PII, only on tarsomeres ta_1_–ta_5_ of PIII. ***Abdomen***: Tergites II–VI with a unique distribution of setae in two longitudinal rows. Tergite IX broadly semicircular, bearing a hump, postero-median and caudal areas with 15 setae mostly located close to base of anal point. Anal point triangular, short and sharply pointed, distinctly curving upwards distally. Sternapodeme orally projecting; phallapodeme unusual comma-like. Virga present, branched apically. Gonocoxite with dorsal distal half parallel-sided; ventral side broadly expanded, bearing several stout setae placed in 2 arched rows. Superior volsella swollen. Inferior volsella subtriangular, inwardly projecting into a spherical lobe, which is hyaline and bare. Gonostylus atypically shaped; globular when viewed dorsally, bean-like in ventral view; crista dorsalis absent; megaseta well developed, tongs-like, visible only in dorsal view.

#### Description.

**Figure 1.**
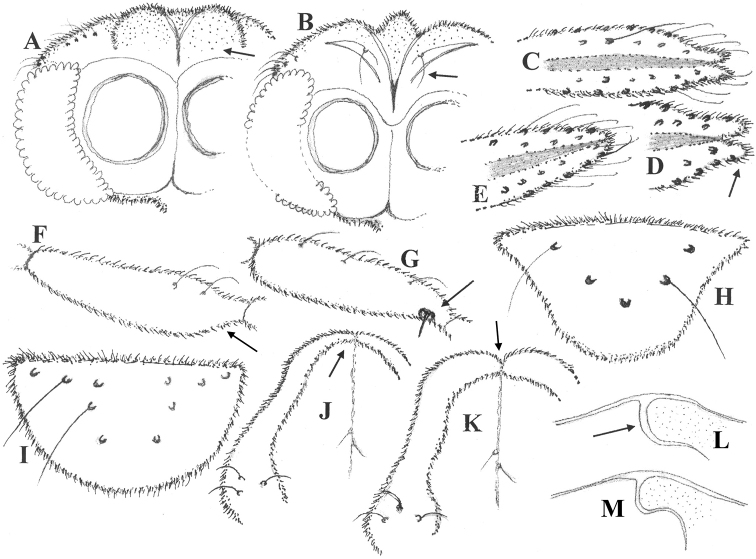
Male imago of *Synorthocladius* spp. Head (dorsal, left side) with vertex, coronal area and temporals of **A***S.federicoi* sp. nov. **B***S.semivirens*. Antenna, apex of last flagellomere of **C, D***S.federicoi* sp. nov. **E***S.semivirens*. Palpomere 3 of **F***S.federicoi* sp. nov. **G***S.semivirens*. Clypeus of **H***S.federicoi* sp. nov. **I***S.semivirens*. Lobes of antepronotum and acrostichals of **J***S.federicoi* sp. nov. **K***S.semivirens*. Humeral area of **L***S.federicoi* sp. nov. **M***S.semivirens*. The arrows indicate some distinguishing characters.

**Figure 2.**
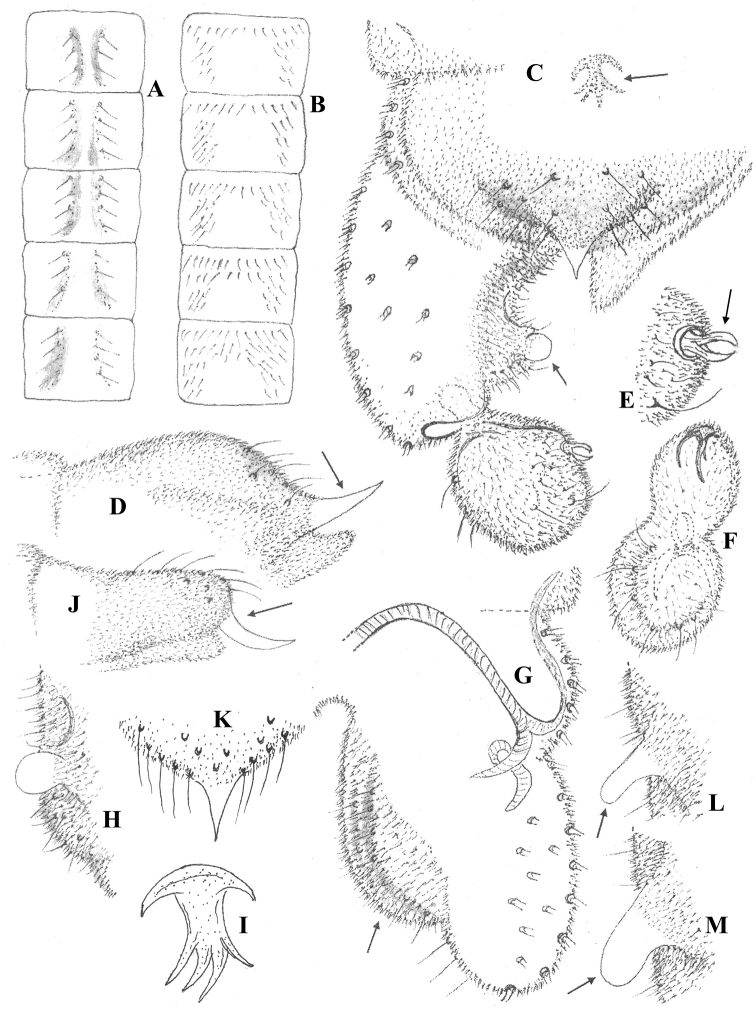
Male imago of *Synorthocladius* spp. Chaetotaxy of tergites II–VI of **A***S.federicoi* sp. nov. **B***S.semivirens*. *S.federicoi* sp. nov. **C** hypopygium in dorsal view **D** tergite IX and anal point in lateral view **E** megaseta, dorsal **F** gonostylus, other aspect in ventral view **G** hypopygium, ventral **H** inferior volsella **I** virga. *S.semivirens***J** tergite IX and anal point in lateral view **K** anal point, dorsal **L, M** inferior volsella, two aspects. The arrows indicate some distinguishing characters.

**Adult male (n = 1; Figs 1A, C–D, F, H, J, L; 2A, C–I).** Medium- to large-sized *Synorthocladius* species. Total length 2.35 mm. Wing length 1.85 mm. TL/WL = 1.27.

***Colouration*.** Blackish species with greenish to brownish legs. Head dark brown including eyes and pedicel; antenna brownish. Thorax with contrasting blackish to dark green mesonotal stripes, area between thoracic stripes greenish; scutellum distinctly contrasting, blackish to brownish. Wing pale brown. Anal segment brown to dark brown with contrasting dark brown to pale gonostylus.

***Head*.** (Fig. 1A). Eyes bare, hairs absent on inner lateral margin; frontal tubercle spherical and well developed; coronal suture reduced, coronal setae absent; temporals 6, uniserial, including 4 inner and 2 outer verticals. Antenna 13-segmented, 790 µm long; last flagellomere (Fig. 1C–D, apical part) 265 µm long, strongly clubbed and bilobed apically, bearing numerous characteristic curved sensilla chaetica; antennal groove begins on segment 3 and reaches the last flagellomere; AR 0.9. Palp 5-segmented, segments 1–2 fused; length (in µm) of segments: 30, 45, 70, 65, 125; palpomere 3 (Fig. 1F) with 2 sensilla clavata, sensilla coeloconica absent. Clypeus (Fig. 1H) inverted safety helmet shaped, with 5 setae in 3 rows.

***Thorax*.** Lobes of antepronotum (Fig. 1J) not gaping and thinner dorsally; acrostichals 2, starting about 150 µm from tip of antepronotum; dorsocentrals 7–8, uniserial; prealars 4; humeral pit absent, notopleural suture (Fig. 1L) with parapsidal fork bent forwards; scutellum with 6 uniserial setae, inserted medially (3 on each side of the midline); preepisternum bare.

***Wing*.** Brachiolum with 1 seta. Number and distribution of setae on veins: R, 5; R_1+2_, 0; R_2+3_, 1; remaining veins bare; squama with 4–5 setae in 1 row.

***Legs*.** Femora of PI and PII subequal, tarsomere ta_5_ of PI–PIII of same size (100 µm long). Tibial spurs present on PI–PIII; length (in µm) of spurs: 50 (PI), 60 (PII), 25 (PIII); pseudospurs absent. Sensilla chaetica present on tibiae and tarsomeres ta_1_–ta_5_ of PI–PII, only present on tarsomeres ta_1_–ta_5_ of PIII. Length (µm) and proportions of legs as in Table 1.

**Table 1.** *Synorthocladiusfedericoi* sp. nov. Length (µm) and proportions of prothoracic (PI), mesothoracic (PII) and metathoracic (PIII) legs.

	fe	ti	ta_1_	ta_2_	ta_3_	ta_4_	ta_5_	LR	BV	SV	BR
PI	625	710	425	355	245	150	100	0.60	2.07	3.14	2.40
PII	645	615	295	175	140	110	100	0.48	2.96	4.27	3.10
PIII	675	745	380	235	190	115	100	0.51	2.81	3.74	2.30

***Abdomen*.** Tergites II–VI (Fig. 2A) with a novel chaetotaxy: two longitudinal rows of setae, 3 to 6 setae on each side of the midline, fewer on tergites V and VI. Hypopygium as in Fig. 2C (dorsal) and G (ventral, with tergite IX, anal point and gonostylus ommited). Tergite IX about 50 µm long and 100 µm maximum width, broadly semi-circular, postero-median and caudal areas with 15 setae (5 located medially, 10 mostly located close to base of anal point); a distinct hump present medio-dorsally, clearly visible in lateral view (Fig. 2D). Anal point (Fig. 2C, D) 25 µm long, 30 µm wide at base, triangular, short and sharply pointed apically, distal part markedly curved upwards (when viewed laterally as in Fig. 2D), basal margin broadly semi-circular. Laterosternite IX with 8 setae (4 on each side). Sternapodeme and phallapodeme as in Fig. 2G, transverse sternapodeme bowed anteriorly; phallapodeme unusual, comma-like. Virga (Fig. 2C, I) well developed and branched apically. Gonocoxite 160 µm long, 80–90 µm wide at base; widest at base and rounded apically; dorsal distal half parallel-sided; ventrally broadly expanded (Fig. 2G), the lobe occupying about 75% of the total length of the gonocoxite, with several stout setae placed in 2 arched rows. Superior volsella swollen. Inferior volsella (Fig. 2C, H) broadly subtriangular at base, inwardly projecting and narrowing into a spherical transparent apex; anterior margin concave, with sclerotization; posterior margin convex, with 3–4 stout setae in 1 row. Gonostylus 55 µm long, 35 µm maximum width, atypically shaped for the genus as shown in Fig. 2C, E, F, globular or bean-like (depending on the angle of view); dorsally (Fig. 2C) with 3–4 stout setae located on distal and lateral parts, anteriorly with distinct sclerotization; ventrally (Fig. 2F) with conspicuous sclerotization anteriorly, with stout setae in a circular row; crista dorsalis absent; megaseta (Fig. 2C, E) 10–12 µm long, tongs-like and well-developed, inserted dorsally halfway from the apex, only visible in dorsal view. HV (total body length divided by length of gonostylus 10 times) = 4.27; HR (length of gonocoxite divided by length of gonostylus) = 2.91.

**Female, pupa and larva**: unknown.

#### Differential diagnosis.

According to Ashe and O’Connor (2012), currently there are six valid *Synorthocladius* species reported from the Palaearctic Region: *S.asamasecundus* Sasa & Hirabayashi, 1991, *S.ginzanpequea* (Sasa & Suzuki, 2001), *S.mongolwexeus* (Sasa & Suzuki, 1997); *S.semivirens*; *S.tamaparvulus* Sasa, 1981 and *S.tusimoijekeus* (Sasa & Suzuki, 1999).

The new species is a *Synorthocladius* based on characters provided in the generic descriptions of Cranston et al (1989) and Liu and Wang (2005): small species (wing length 1.85 mm); antenna with 13 flagellomeres, with groove beginning on flagellomere 3, apical flagellomere with characteristic curved sensilla chaetica, antennal ratio less than 1 (0.9); eyes bare, temporal setae few (6), uniserial; antepronotal lobes fused medially, acrostichals few (2), dorsocentrals and scutellars uniserial; wing membrane without setae, squama with sparse setal fringe (4/5); anal point short and without setae; inferior volsella bilobed. However, *S.federicoi* sp. nov. is very different from previously described species in the following respects:

- Frontal tubercles broadly globular (Fig. 1A); indistinct in S. semivirens (Fig. 11B), absent in S. tamaparvulus (Sasa 1981, fig. 11B);
- Inner temporals of 4 setae in 1 row (Fig. 1A); with a single seta in S. semivirens (Fig. 1B);
- Last flagellomere of antenna distinctly bilobed apically (Fig. 1C, D); rounded and simply clubbed in S. semivirens (Fig. 1E) and S. tamaparvulus (Sasa 1981, fig. 11C);
- Lobes of antepronotum not gaping (Fig. 1J); gaping in S. semivirens (Fig. 1K);
- Notopleural suture with parapsidal branch arched (Fig. 1L); sinuate in S. semivirens (Fig. 1M);
- Unusual pattern of setae on tergites II–VI (Fig. 2A); more generally distributed in S. semivirens (Fig. 2B) and S. tamaparvulus (Sasa 1981, fig. 11F);
- Tergite IX with a distinct hump (Fig. 2D); linearly elongate in S. semivirens (Fig. 2J);
- Basal part of anal point semi-circular and slightly bent downwards (Fig. 2C, dorsal; Fig. 2D, lateral); sub-circular and strongly projecting downwards in S. semivirens (Fig. 2K, dorsal; Fig. 2J, lateral);
- Virga branched (Fig. 2C, I); absent in S. semivirens and S. tamaparvulus;
- Inferior volsella broadly subtriangular basally, narrowing towards apex and ending in a unique (for the genus) spherical lobe; elongate finger-like to nose-like in both S. semivirens (Fig. 2L, M) and S. tamaparvulus (Sasa 1981, figs 12A, E);
- Gonostylus unusual in shape (globular or bean-like as in Fig. 2C, E, F); elongate and more or less parallel-sided in S. semivirens and S. tamaparvulus, as illustrated by Cranston et al. (1989, fig. 9.83E), Liu and Wang (2005, figs 4, 8), Langton and Pinder (2007, fig. 192D) and Sasa (1981, fig. 12A, E).

### Key to adult males of known *Synorthocladius* species from Europe

**Table:** 

1	Inferior volsella with spherical apex (Fig. 2C, H); last flagellomere of antenna bilobed apically (Fig. 1C, D); gonostylus globular dorsally (Fig. 2C, E).	***S.federicoi* sp. nov.**
–	Inferior volsella finger-like or nose-like (Fig. 2L, M); last flagellomere of antenna simply clubbed (Fig. 1E); gonostylus slender as in Sasa (1981, fig. 12E), Liu and Wang (2005, figs 4, 8), Langton and Pinder (2007, fig. 192D).	*** S. semivirens ***

## Discussion

The newly described species can be considered as a local biogeographic representative of the Venetian Pre-Alps. Consequently, the description here of *S.federicoi* sp. nov. increases the total number of valid species of *Synorthocladius* from Europe to two.

**Figure 3.**
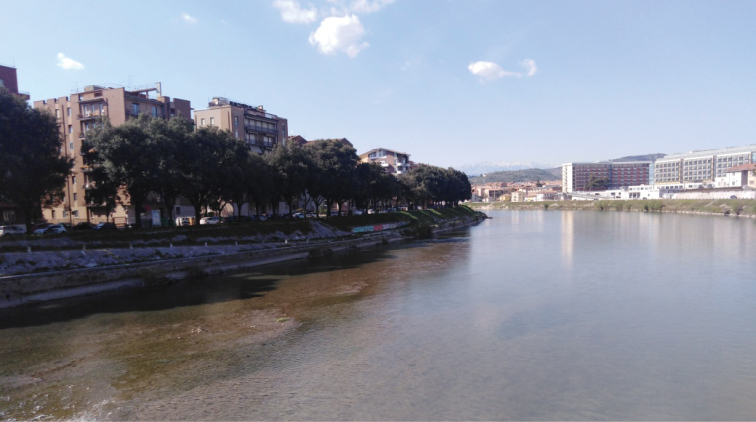
Type locality of *Synorthocladiusfedericoi* sp. nov., Adige River, Verona (northern Italy) (by V. Lencioni).

Larvae of *Synorthocladius* species are typically rheobiontic, occurring especially in rheocrene mountain springs and streams fed by groundwater (krenal) (Reiss 1968, 1989; Evrard 1995; Lindegaard 1995; Lencioni et al. 2011, 2012, 2018; Kettani and Moubayed-Breil 2018; Murray et al. 2018), but also in the rhithral and potamal reaches of rivers with high current velocity (Rossaro 1982). The holotype and only known specimen of the new species was collected in a moderately shaded lotic habitat with sandy to gravely substrate supplied by fresh groundwater, which maintains a low annual variation of temperature. The type locality (Fig. 3) is in the hyporhithral sector of the Adige River (Braioni and Ruffo 1986). It includes stones covered by submerged and emerged bryophytes and microalgae, which provide favourable microhabitats for chironomid larval stages. The environmental data of water recorded in the type locality are: conductivity = 262 µS/cm; pH = 8.4; temperature = 12.5 °C. Emergence of adult chironomids is usually observed in early spring (March–April).

*Synorthocladiusfedericoi* sp. nov. is known only from its type locality in the Venetian Pre-Alps (a mountain range of the Italian Alps). It would appear to be a biogeographic representative of lotic habitats delimited by the south-eastern part of the Italian Alps. It is likely to be more widespread in similar lotic habitats or Alpine streams of northern Italy.

Chironomid species encountered with *S.federicoi* sp. nov. include: *Conchapelopiapallidula* (Meigen, 1818), *C.melanops* (Meigen, 1818), *Paramerinacingulata* (Walker, 1856), *Cardiocladiusfuscus* Kieffer, 1924, *Cricotopusannulator* Goetghebuer, 1927, *C.levantinusoccidentalis* Moubayed-Breil & Ashe, 2011, *C.tremulus* (Linnaeus, 1758), *Eukiefferielladevonica* (Edwards, 1929), *E.ilkleyensis* (Edwards, 1929), *E.lobifera* Goetghebuer, 1934, *Paracricotopusniger* (Kieffer, 1913), *Parametriocnemusstylatus* (Spärck, 1923), *Rheocricotopuschalybeatus* (Edwards, 1929), *Synorthocladiussemivirens* (Kieffer, 1909), *Tveteniacalvescens* (Edwards, 1929), *Micropsectraatrofasciata* (Kieffer, 1911) and *Rheotanytarsuscurtistylus* (Goetghebuer, 1921).

## Supplementary Material

XML Treatment for Synorthocladius

XML Treatment for Synorthocladius federicoi
